# Supplementary material for: Difference in Incontinence Pad Use between Patients after Radical Prostatectomy and Cancer-Free Population with Subgroup Analysis for Open vs. Minimally Invasive Radical Prostatectomy: A Descriptive Analysis of Insurance Claims-Based Data
Source: Int J Environ Res Public Health. 2021 Jun 27;18(13):6891. doi: 10.3390/ijerph18136891 (PMC8296932; doi:10.3390/ijerph18136891)
Supplement: Supplementary file 1 [file ijerph-18-06891-s001.zip › S1 Methods and Materials.pdf]

S1 Methods and Materials

We first identified dates and specific diagnoses using ICD codes for PCa (ICD-9 Code: 185, ICD-10: C61), other cancer (ICD C00–C97), and BPH (ICD-9: 600, ICD-10: N40). Then, surgical procedures treating PCa and BPH were characterized using MEL codes for RP (MEL code: "JG050","JG060","JG070","JG080") and transurethral resection of the prostate (TURP) (MEL code: ”JG020”), respectively. Based on these characteristics, participants were categorized into one of the following three groups: 1) men with PCa and underwent RP: diagnosis of PCa; date of RP between 2013-2015, and age at RP ≥ 45 years; 2) cancer-free men with non-operated BPH: no diagnosis of any cancer; diagnosis of BPH between 2013-2015, did not undergo TURP; 3) cancer-free men without BPH: a randomly selected group of patients whose hospital stays were documented between 2013-2015; no PCa or BPH diagnoses documented; age at 1/1/2014 was ≥ 45 years.
