# Supplementary material for: Difference in Incontinence Pad Use between Patients after Radical Prostatectomy and Cancer-Free Population with Subgroup Analysis for Open vs. Minimally Invasive Radical Prostatectomy: A Descriptive Analysis of Insurance Claims-Based Data
Source: Int J Environ Res Public Health. 2021 Jun 27;18(13):6891. doi: 10.3390/ijerph18136891 (PMC8296932; doi:10.3390/ijerph18136891)
Supplement: Supplementary file 1 [file ijerph-18-06891-s001.zip › S1 Supplementary Table.pdf]

S1 Supplementary Table: Poisson regression modelling post-operative monthly rate of prescribed incontinence pads adjusting for age group (45-<60, 60-<70, 70+) and pre-operative rates of prescribed incontinence pads by RP procedures.

	RP-Open				RP-minimally invasive			
Parameter	Estimate	Low 95%CI	Upp 95%CI	p-value	Estimate	Low 95%CI	Upp 95%CI	p-value
Intercept	4.0904	3.7022	4.4786	<.0001	5.2858	5.0498	5.5218	<.0001
Rate_before	17.7992	13.9108	21.6877	<.0001	3.0723	1.7689	4.3757	<.0001
Age [Ref: 45-<60]								
60-<70	-0.8483	-1.4387	-0.2579	0.0049	0.341	0.0572	0.6248	0.0185
70+	-0.3273	-1.0173	0.3628	0.3526	0.5344	0.2224	0.8464	0.0008
