# Supplementary figures and images for: Difference in Incontinence Pad Use between Patients after Radical Prostatectomy and Cancer-Free Population with Subgroup Analysis for Open vs. Minimally Invasive Radical Prostatectomy: A Descriptive Analysis of Insurance Claims-Based Data
Source: Int J Environ Res Public Health. 2021 Jun 27;18(13):6891. doi: 10.3390/ijerph18136891 (PMC8296932; doi:10.3390/ijerph18136891)

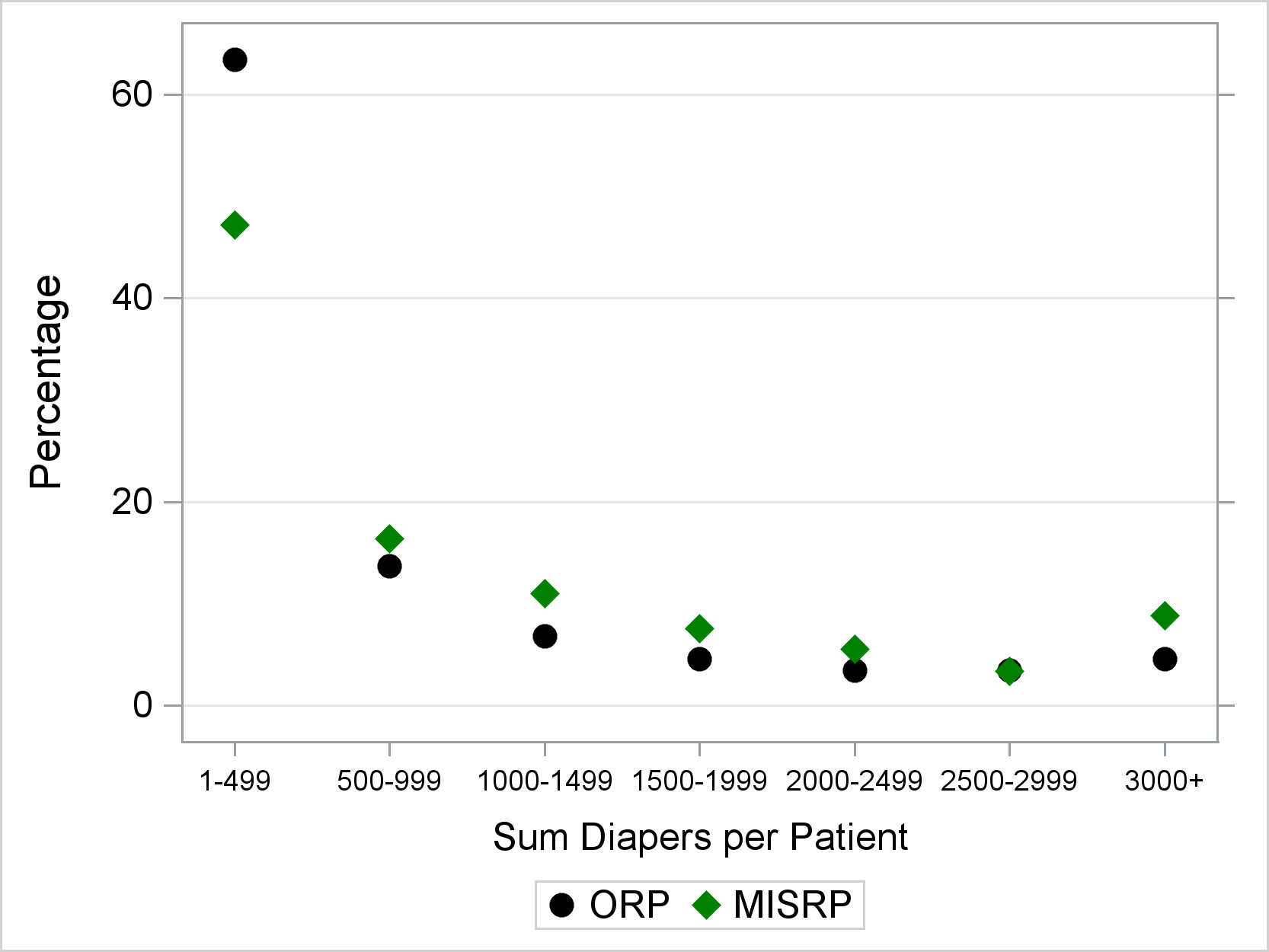

Supplement: Supplementary file 1 [file ijerph-18-06891-s001.zip › S1 Suppl_Figure_1.png]
